# Development and testing of a novel survey to assess Stakeholder-driven Community Diffusion of childhood obesity prevention efforts

**DOI:** 10.1186/s12889-018-5588-1

**Published:** 2018-05-31

**Authors:** Ariella R. Korn, Erin Hennessy, Ross A. Hammond, Steven Allender, Matthew W. Gillman, Matt Kasman, Jaimie McGlashan, Lynne Millar, Brynle Owen, Mark C. Pachucki, Boyd Swinburn, Alison Tovar, Christina D. Economos

**Affiliations:** 1 Friedman School of Nutrition Science and Policy, Tufts University, 150 Harrison Ave., Boston, MA 02111 USA; 2 The Brookings Institution, 1775 Massachusetts Ave., NW, Washington, DC 20036 USA; 3 Global Obesity Centre (GLOBE), Deakin University, 1 Gheringhap St, Geelong, VIC 3220 Australia; 4 Division of Chronic Disease Research Across the Lifecourse, Department of Population Medicine, Harvard Medical School and Harvard Pilgrim Health Care Institute, Landmark Center, 401 Park Drive, Suite 401 East, Boston, MA 02215 USA; 5 Department of Sociology, University of Massachusetts Amherst, 200 Hicks Way, Thompson Hall 532, Amherst, MA 01003 USA; 6 School of Population Health, University of Auckland, Private Bag 92019, Auckland, 1142 New Zealand; 7 Department of Nutrition and Food Sciences, University of Rhode Island, 125 Fogarty Hall, Kingston, RI 02881 USA

**Keywords:** Community-based interventions, Community engagement, Childhood obesity prevention, Survey development

## Abstract

**Background:**

Involving groups of community stakeholders (e.g., steering committees) to lead community-wide health interventions appears to support multiple outcomes ranging from policy and systems change to individual biology. While numerous tools are available to measure stakeholder characteristics, many lack detail on reliability and validity, are not context specific, and may not be sensitive enough to capture change over time. This study describes the development and reliability of a novel survey to measure Stakeholder-driven Community Diffusion via assessment of stakeholders’ social networks, knowledge, and engagement about childhood obesity prevention.

**Methods:**

This study was completed in three phases. Phase 1 included conceptualization and online survey development through literature reviews and expert input. Phase 2 included a retrospective study with stakeholders from two completed whole-of-community interventions. Between May–October 2015, 21 stakeholders from the Shape Up Somerville and Romp & Chomp interventions recalled their social networks, knowledge, and engagement pre-post intervention. We also assessed one-week test-retest reliability of knowledge and engagement survey modules among Shape Up Somerville respondents. Phase 3 included survey modifications and a second prospective reliability assessment. Test-retest reliability was assessed in May 2016 among 13 stakeholders involved in ongoing interventions in Victoria, Australia.

**Results:**

In Phase 1, we developed a survey with 7, 20 and 50 items for the social networks, knowledge, and engagement survey modules, respectively. In the Phase 2 retrospective study, Shape Up Somerville and Romp & Chomp networks included 99 and 54 individuals. Pre-post Shape Up Somerville and Romp & Chomp mean knowledge scores increased by 3.5 points (95% CI: 0.35–6.72) and (− 0.42–7.42). Engagement scores did not change significantly (Shape Up Somerville: 1.1 points (− 0.55–2.73); Romp & Chomp: 0.7 points (− 0.43–1.73)). Intraclass correlation coefficients (ICCs) for knowledge and engagement were 0.88 (0.67–0.97) and 0.97 (0.89–0.99). In Phase 3, the modified knowledge and engagement survey modules included 18 and 25 items, respectively. Knowledge and engagement ICCs were 0.84 (0.62–0.95) and 0.58 (0.23–0.86).

**Conclusions:**

The survey measures upstream stakeholder properties—social networks, knowledge, and engagement—with good test-retest reliability. Future research related to Stakeholder-driven Community Diffusion should focus on prospective change and survey validation for intervention effectiveness.

**Electronic supplementary material:**

The online version of this article (10.1186/s12889-018-5588-1) contains supplementary material, which is available to authorized users.

## Background

Community-based interventions have demonstrated effective childhood obesity prevention at the population level [1–3]. In particular, “whole-of-community” interventions are recommended in which entire communities are exposed to programs, policies, and environments intended to reduce obesity risk [4–9]. Successful whole-of-community interventions necessitates the recognition of complex organizational and community dynamics and the influence of community leaders and stakeholders (hereafter referred to as stakeholders) from various sectors to build capacity, enhance community well-being, and promote systems change [2, 10–16].

Understanding the upstream processes by which investigators and stakeholder groups (e.g., coalitions, steering committees) conceive, design, implement, and adapt whole-of-community interventions is a critical step to inform prevention efforts and impact research outcomes [17, 18]. Extant tools to measure stakeholder characteristics, such as empowerment [19], collaboration [20], and readiness for change [21] have notable weaknesses that limit utility, such as lack of detail on reliability and validity and/or are not context specific. Sensitive, reliable, and valid tools to measure longitudinal information on context, including differences in stakeholder social networks and diffusion of information, are needed to shift how investigators approach, understand, and work with community partners. This may contribute to the widespread adoption and scaling of the whole-of-community model to improve population health [18].

The Childhood Obesity Modeling for Prevention And Community Transformation (COMPACT) study funded by the National Institutes of Health (R01HL115485; 2013–2018) seeks to apply systems methods to better understand stakeholders’ leadership roles in whole-of-community interventions [22]. We hypothesize that stakeholder groups may be a driving factor in the success of interventions through a process of “Stakeholder-driven Community Diffusion”. As an initial test of this theory, an agent-based model has been designed to understand how stakeholders (the agents) involved in completed and ongoing whole-of-community interventions in the US, Australia, and New Zealand use their social networks to diffuse their knowledge about and engagement with childhood obesity prevention efforts. This work, however, is also dependent upon reliable and valid measurement of stakeholder characteristics. Therefore, this paper describes the development and reliability testing of the COMPACT Stakeholder-driven Community Diffusion Survey, a unique multi-method survey that allows quantification of changes in social networks, knowledge, and engagement properties of stakeholders involved in whole-of-community interventions.

## Methods and results

**Fig. 1:**
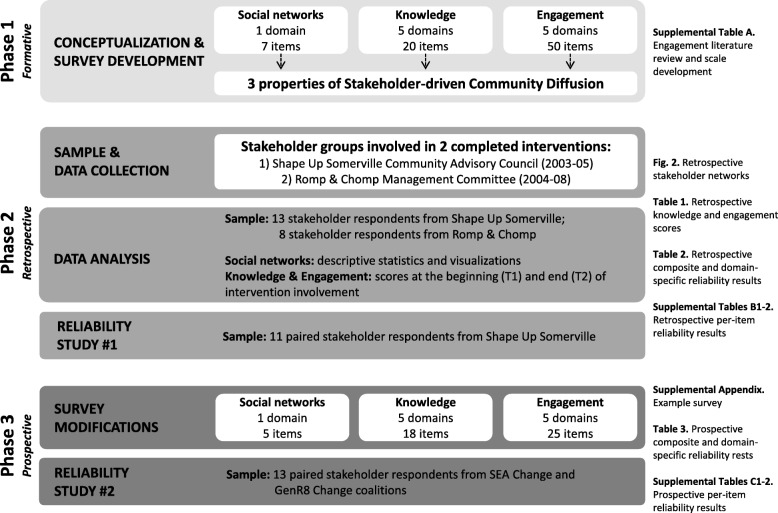
Overview of the development and reliability testing of the COMPACT Stakeholder-driven Community Diffusion Survey

This study was completed in three phases (Fig. 1). Methods and results are reported below by study phase. Phase 1 included conceptualization and survey development assessed for content validity. Phase 2 included a pre-post assessment and reliability testing (test-retest) with stakeholders from two completed whole-of-community interventions using retrospective reporting [23]. Phase 3 included survey modifications and a second prospective reliability assessment.

### Phase 1: Survey development

The COMPACT Stakeholder-driven Community Diffusion Survey included three modules to (a) assess the network structure of stakeholders’ professional relationships related to childhood obesity prevention efforts, (b) knowledge about childhood obesity prevention, and (c) engagement with the issue.

### Part A: Social networks

In Stakeholder-driven Community Diffusion, social networks represent pathways for knowledge and engagement diffusion. The survey was designed to allow respondents to name up to 20 people with whom they had “discussed issues related to childhood obesity” during an intervention [24]. Due to the retrospective nature of the initial survey and to diminish likelihood of inaccurate recall, we used two name generation methods (free recall and a roster of stakeholders who had already provided informed consent for the survey) in a three-stage procedure (free recall, roster identification, final free recall) [25]. This approach was used to capture the complete network of bounded stakeholder groups (e.g., steering committees) and stakeholders’ broader networks when exploring community-wide connections [25].

### Part B: Knowledge

We conceptualized knowledge as stakeholders’ understanding of community-wide efforts to prevent childhood obesity. We identified five domains from completed intervention trials that reduced unnecessary weight gain among children [6, 7, 26]:

1. The problem of childhood obesity (“Problem”)
2. Modifiable determinants of childhood obesity and level of social ecology to address them, e.g., individual behavior change versus environment and policy change (“Intervention factors”)
3. Stakeholders’ roles in the whole intervention, what others are doing, and knowledge of multi-setting components (“Roles”)
4. How to intervene to achieve sustainability (“Sustainability”)
5. Available resources (“Resources”)

We conducted comprehensive literature reviews (peer-reviewed and grey) to source relevant instruments and survey items measuring aspects of community readiness, group dynamics, coalitions, and community-based participatory research (CBPR) to adapt and apply to the identified domains. For “resources”, we adapted four items from the Community Capacity Index [27] and the Community Readiness Handbook [21]. For the remaining domains, we identified eight items from the CBPR Conceptual Model matrix of variables and instruments [12, 17, 28] and the coalition literature. Five research team members with experience in community-based interventions scored items to assess content validity. Scoring resulted in disagreement on items to include. Through iterative critique and feedback, the team developed new fact-based, multiple-choice items for domains 1–4 (four items per domain). The knowledge domain included 20 total items.

### Part C: Engagement

We conceptualized engagement as a latent construct representing stakeholders’ enthusiasm and agency for preventing childhood obesity in their community. Our Stakeholder-driven Community Diffusion theory suggests that engagement motivates stakeholders to share their knowledge with others, and represents stakeholders’ desires and ability to translate their knowledge into effective action for whole-of-community interventions.

We used the CBPR Model to identify domains describing stakeholder engagement [12, 28]:

1. Exchange of skills and understanding (“Dialogue and mutual learning”)
2. Willingness to compromise and adapt (“Flexibility”)
3. Ability or capacity to have an effect on course of events, others’ thinking, and behavior (“Influence and power”)
4. Action of directing and being responsible for a group of people or course of events (“Leadership and stewardship”)
5. Belief and confidence in others (“Trust”)

We used 46 items from existing instruments cited in the CBPR Model to construct an engagement scale [17]. We also conducted a secondary search in Scopus, PubMed, and the National Cancer Institute’s Team Science Toolkit [29] for community and group partnership tools, yielding 104 total items from 20 instruments.

Six research team members evaluated the 104 items for content validity. They scored items from 0 to 2 points (0 = no; 1 = maybe; 2 = yes), with a maximum per-item score of 12 points. Item scores ranged from 3 to 11 points (mean = 7.2; SD = 1.8). We eliminated items with low scores (≤ 6 points; *n* = 37) and/or if an item scored lower than a similar item. We retained 50 items from 17 instruments: dialogue and mutual learning (11), flexibility (8), influence and power (4), leadership and stewardship (22), and trust (5) (Additional file 1: Table S1A). We set response options to a 5-point agree/disagree Likert scale and adapted wording to fit the context of whole-of-community childhood obesity prevention interventions.

### Phase 2: Retrospective study

#### Methods

##### *Participants*

Respondents were members of stakeholder groups involved in two completed whole-of-community childhood obesity interventions: Shape Up Somerville (SUS) [6] and Romp & Chomp (R&C) [7]. Both interventions demonstrated measured reductions in childhood obesity prevalence. SUS was a community-based environmental change intervention from 2003 to 2005 targeting early elementary school children in Somerville, Massachusetts, USA. The SUS Community Advisory Council included stakeholders from academia, public schools, foodservice, local health department, community-based organizations, and met every 2–4 months throughout the intervention. R&C was a capacity-building and environmental intervention from 2004 to 2008 targeting children from birth to five years in Geelong, Victoria, Australia. The R&C Management Committee [30] included stakeholders from academia, local health department, government, department of human services, and the local kindergarten association, and met every 1–2 months.

### Procedures

We identified potential participants’ names from historical SUS and R&C records and meeting minutes, and then acquired current contact information (email and/or telephone) via records, existing contacts, and the internet. We first contacted participants for informed consent. Upon providing consent, we invited participants to complete the web-based (Qualtrics) survey in May–June 2015 for SUS and August–October 2015 for R&C.

To aid participants’ memories in what life was like during the interventions, the surveys started by listing historical milestones at the local, state, and national level (e.g., elected government officials, major sports victories). This was followed by an optional, open-ended question that asked participants to “write any names, phrases, or keywords that describe what was going on in your life” during the intervention period. We informed participants that this response would not be retained and that the purpose was to help them provide more accurate recalled responses [24].

We then asked participants to identify social relationships and to report their own levels of knowledge and engagement related to childhood obesity prevention at the start (T1) and end (T2) of their involvement in SUS or R&C. Time was based on intervention involvement due to varying participation and attrition in stakeholder meetings. Participants reported their gender, current age, education, and affiliated organizational sector (e.g., school administration) at the start of the intervention.

To assess the test-retest reliability of the knowledge and engagement survey components, we asked participants to complete a second web-based survey, one week after the first survey.

In the SUS study, we offered participants up to $49 (electronic Amazon gift card) for completing both test-retest surveys. Consistent with usual practices in Australian studies of this type, no monetary incentive was offered to R&C participants. Procedures for individuals participating in research were approved by the Tufts University Institutional Review Board and the Deakin University Human Ethics Advisory Group for the SUS and R&C studies, respectively.

### Data analysis

#### Demographics

We calculated frequencies for categorical variables (gender, education, organizational sector affiliation) and means and standard deviations (SD) for participant age.

#### Social networks

We extracted online data from the three-stage name generator of childhood obesity ‘discussion’ networks and imported to the [sna], [igraph], and [network] packages in the R programming language to conduct descriptive analyses and produce sociograms [31–33]. In the sociograms, participants are represented as nodes and are connected by a directed tie to represent a discussion relationship. Visualizations demonstrate structural attributes of networks and are useful in generating hypotheses about pathways for knowledge and engagement diffusion. Calculated descriptive connectivity statistics included number of nodes and ties, density (the proportion of ties to the number of possible ties between node pairs), and in-degree centralization (an indicator of node connectivity, or the extent to which one or few nodes in the network receive a high number of ties).

### Knowledge and engagement

We calculated composite and domain-specific scores (mean, SD) at both time points. Knowledge domains 1–4 each had four multiple-choice questions with a maximum score of four points per domain (− 1 = incorrect response; 0 = not sure; 1 = correct response). Knowledge domain 5 had four 4-point agree/disagree Likert-scale items (− 1 = strongly disagree; − 0.5 = disagree; 0.5 = agree; 1 = strongly agree) with a maximum score of 4 points. The maximum composite score was 20 points. There were 50 5-point agree/disagree Likert-scale engagement items. We weighted scores based on number of items per domain to ease domain-domain comparisons, with a maximum composite score of 25 points (1 = strongly disagree to 5 = strongly agree). We used paired t-tests and corresponding 95% CIs to assess change in mean knowledge and engagement scores from T1 to T2 within interventions (test survey data used).

### Knowledge and engagement reliability

We analyzed reliability data from T1. We assessed item-specific test-retest reliability using Cohen’s weighted Kappa statistic (κ_w_) [34]. We calculated intraclass correlation coefficients (ICCs) and within-subject coefficients of variation (WSCV), each with 95% confidence intervals (CIs), to inform composite and domain-specific reliability. We used Cronbach’s alpha (α) to assess composite and domain-specific engagement internal scale consistency. We did not calculate scale consistency for the retrospective knowledge measure, as items in domains 1–4 assessed fact-based knowledge and were not expected to relate. Data were analyzed using SAS 9.3 (Cary, NC) and StataSE 14 (College Station, TX).

## Results

### Sample characteristics

From historical records, we identified 25 SUS stakeholders and acquired contact information for 23, of which 15 provided consent (65.2%). Consenting participants’ names were included in the network roster. Thirteen participants completed the first reliability survey (56.5%). For R&C, we identified 21 stakeholders and acquired contact information for 12. Eleven provided consent (91.7%) and were included in the network roster. Eight participants completed the first survey (66.7%).

Most SUS and R&C respondents were female (*n* = 11; 84.6% and *n* = 5; 62.5%). At T1, mean ages were 40.9 (SD = 9.7) and 41.4 (SD = 10.8) years for SUS and R&C, respectively. The majority of SUS and R&C respondents had a Bachelor’s degree or higher (*n* = 13; 100% and *n* = 7; 87.5%). SUS respondents reported affiliations with university/academia (*n* = 4; 30.8%), community-based organizations (*n* = 4; 30.8%), school administration (*n* = 1; 7.7%), afterschool programs (*n* = 2; 15.4%), and local health department (*n* = 2; 15.4%). R&C respondents represented university/academia (*n* = 5; 62.5%), community-based organizations (*n* = 1; 12.5%), and local government (*n* = 2; 25.0%).

### Social networks

**Fig. 2:**
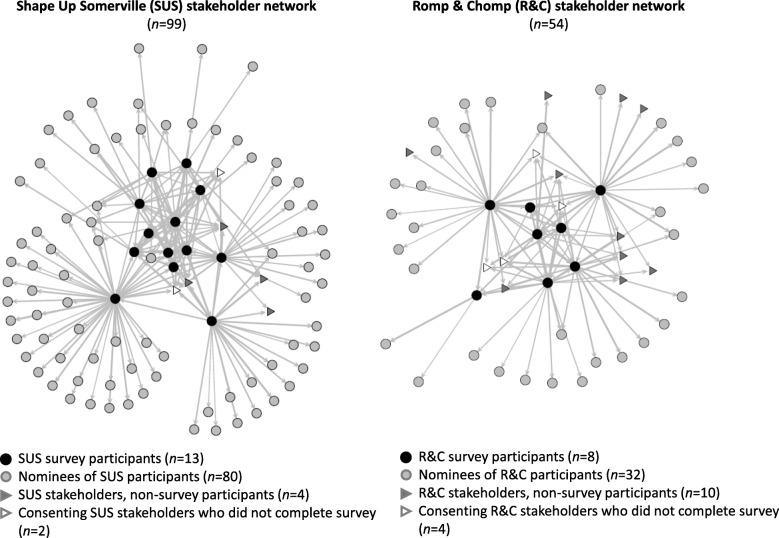
Phase 2 stakeholder networks from the Shape Up Somerville and Romp & Chomp interventions. Data are from the three-stage name generator of childhood obesity discussion networks. Visualizations are for illustrative purposes only and were not used to interpret results or draw conclusions

The SUS and R&C stakeholder networks are shown in Fig. 2. The SUS network had 99 nodes (individuals), 218 ties (relationships between individuals), density of 0.02 (proportion of ties to the total number of possible ties between node pairs), and in-degree centralization of 0.09 (the extent to which one or few nodes receive a high number of ties). The R&C network had 54 nodes, 126 ties, a density of 0.04, and in-degree centralization of 0.07. Readers are referred to McGlashan et al. for further description of SUS and R&C stakeholder networks [24].

### Knowledge and engagement scores

Mean composite and domain-specific scores are reported in Table 1. Of 20-points maximum, the mean SUS composite knowledge score increased from 10.4 points (SD = 5.2) at T1 to 13.9 points (SD = 3.8) at T2 (3.5 points; 95% CI: 0.35–6.72). The mean composite knowledge R&C score increased from 10.1 points (SD = 6.3) at T1 to 13.6 points (SD = 2.7) at T2 (3.5 points; 95% CI: -0.42-7.42). Mean engagement scores were similar among SUS and R&C respondents at T1 and T2.

**Table 1:** Phase 2 knowledge and engagement scores at the start and end of stakeholders’ intervention involvement

Constructs and domains	# items	Max. score	Shape Up Somerville (*n* = 13)			Romp & Chomp (*n* = 8)		
			Mean score (SD)		T2-T1 difference (95% CI)^a^	Mean score (SD)		T2-T1 difference (95% CI)^a^
			T1	T2		T1	T2	
Knowledge^b^								
Composite	20	20	10.38 (5.16)	13.92 (3.78)	3.54 (0.35–6.72)*	10.13 (6.28)	13.63 (2.68)	3.50 (−0.42–7.42)
Domain-specific								
1. Problem	4	4	3.23 (1.24)	3.77 (0.60)	0.54 (−0.23–1.30)	3.13 (1.36)	4.00 (0.00)	0.88 (−0.26–2.01)
2. Intervention factors	4	4	0.92 (2.06)	2.54 (1.45)	1.62 (0.50–2.73)*	1.50 (2.00)	1.88 (1.36)	0.38 (−0.96–1.71)
3. Roles	4	4	2.31 (1.93)	3.38 (1.26)	1.08 (−0.07–2.22)	1.63 (2.00)	3.38 (0.92)	1.75 (−0.13–3.63)
4. Sustainability	4	4	2.46 (1.39)	2.46 (1.20)	0.00 (−0.55–0.55)	2.88 (1.25)	2.75 (1.39)	−0.13 (−1.07–0.82)
5. Resources	4	4	1.46 (1.74)	2.09 (1.83)^d^	0.73 (− 0.64–2.09)^d^	1.00 (1.25)	1.63 (0.79)	0.63 (0.00–1.25)*
Engagement^c^								
Composite	50	25	17.89 (3.28)	18.98 (3.43)	1.09 (−0.55–2.73)	19.02 (2.11)	19.67 (1.52)	0.65 (−0.43–1.73)
Domain-specific								
1. Dialogue & mutual learning	11	5	3.99 (0.75)	4.29 (0.54)	0.29 (−0.04–0.62)	3.93 (0.49)	3.98 (0.40)	0.05 (−0.13–0.22)
2. Flexibility	8	5	3.68 (0.71)	3.66 (1.16)	−0.02 (− 0.70–0.66)	3.89 (0.28)	3.94 (0.27)	0.05 (− 0.05–0.14)
3. Influence & power	4	5	3.12 (0.81)	3.42 (0.88)	0.31 (0.02–0.59)*	3.47 (0.67)	3.66 (0.50)	0.19 (−0.12–0.50)
4. Leadership & stewardship	22	5	3.60 (0.71)	3.84 (0.69)	0.23 (−0.12–0.58)	3.78 (0.44)	3.88 (0.36)	0.10 (−0.19–0.38)
5. Trust	5	5	3.78 (0.78)^e^	4.08 (0.81)^e^	0.30 (− 0.07–0.67)^e^	3.95 (0.67)	4.23 (0.46)	0.28 (−0.05–0.60)

*Notes*: T1 and T2 are the start and end, respectively, of stakeholders’ intervention involvement. CI = confidence interval. *p < 0.05

^a^Paired t-test

^b^Knowledge items for domains 1–4 were multiple choice or true/false with the following scoring: − 1 = incorrect; 0 = not sure; 1 = correct. Items for domain 5 were on a 4-point agree/disagree Likert scale with the following scoring (to remain consistent with domains 1–4 scores): − 1 = strongly disagree; − 0.5 = disagree; 0.5 = agree; 1 = strongly agree

^c^Engagement items were on a 5-point agree/disagree Likert scale. Data were weighted to reflect the number of items per domain to ease domain-to-domain comparisons. Composite scores are a mean of the total, not a sum of means; therefore, domain scores may not add up to composite score

^d^*n* = 11; difference and 95% CI calculated from paired respondents

^e^*n* = 12

### Knowledge and engagement reliability

SUS test-retest reliability data are presented, but not from R&C due to low retest sample size (*n* = 6). Eleven of 13 SUS respondents completed the one-week retest survey (84.6%). Composite and domain-specific results are shown in Table 2, while per-item results are available in Additional file 2: Table S2B1-B2. The ICC and WSCV for composite knowledge were 0.88 (95% CI: 0.67–0.97) and 0.06 (95% CI: 0.04–0.10), respectively. The ICC and WSCV for composite engagement were 0.97 (95% CI: 0.89–0.99) and 0.04 (95% CI: 0.03–0.07). Across test-retest surveys, the average Cronbach’s α for composite engagement scale consistency was 0.99.

**Table 2:** Phase 2 reliability results (*n* = 11 paired responses; Shape Up Somerville Community Advisory Council members)

Constructs and domains	# items	One-week test-retest reliability^a^		Internal scale consistency (Cronbach’s α)^b^		
		ICC (95% CI)	WSCV (95% CI)	*Test*	*Retest*	Average
Knowledge				**–**	**–**	**–**
Composite	20	0.88 (0.67–0.97)	0.06 (0.04–0.10)			
Domain-specific						
1. Problem	4	0.08 (0.00–1.00)	0.14 (0.09–0.23)	**–**	**–**	**–**
2. Intervention factors	4	0.83 (0.55–0.95)	0.19 (0.11–0.33)	**–**	**–**	**–**
3. Roles	4	0.76 (0.44–0.93)	0.15 (0.09–0.25)	**–**	**–**	**–**
4. Sustainability	4	0.70 (0.34–0.91)	0.13 (0.08–0.21)	**–**	**–**	**–**
5. Resources	4	0.59 (0.21–0.88)	0.23 (0.13–0.40)	**–**	**–**	**–**
Engagement						
Composite	50	0.97 (0.89–0.99)	0.04 (0.03–0.07)	0.98	0.99	0.99
Domain-specific						
1. Dialogue & mutual learning	11	0.96 (0.86–0.99)	0.05 (0.03–0.08)	0.95	0.97	0.96
2. Flexibility	8	0.86 (0.61–0.96)	0.10 (0.06–0.16)	0.92	0.95	0.94
3. Influence & power	4	0.88 (0.67–0.97)	0.15 (0.09–0.26)	0.91	0.95	0.93
4. Leadership & stewardship	22	0.97 (0.90–0.99)	0.04 (0.03–0.07)	0.97	0.98	0.98
5. Trust	5	0.93 (0.78–0.98)	0.07 (0.04–0.11)	0.94	0.97	0.96

*ICC* intraclass correlation coefficient, *WSCV* within-subject coefficient of variation, *CI* confidence interval

^a^Reliability results from T1, i.e., the start of Community Advisory Council members’ involvement in the Shape Up Somerville intervention

^b^Internal scale consistency was not calculated for the retrospective knowledge survey component. Items were fact-based (multiple choice or true/false), and therefore not expected to relate to each other

### Phase 3: Prospective study

#### Methods

##### *Survey modifications*

We modified the retrospective survey to evaluate whole-of-community childhood obesity prevention interventions prospectively (Additional file 3: Appendix). The social network name generator was limited to free recall in anticipation of prospectively assessing new stakeholder networks, in which names were not yet known to populate a roster. Items were changed from past to present tense. Multiple-choice and true/false knowledge items with “correct” answers (domains 1–4) were adapted to fit a 5-point Likert agree/disagree scale. This change was implemented to capture greater variability in scaled responses over time (for future survey applications) and to include a neutral response option, consistent with engagement items. Domain 5 knowledge items were changed from a 4- to 5-point Likert scale for consistency with other items. The engagement scale was reduced from 50 to 25 items based on Phase 2 κ_w_ values (0.3 cut-off) and expert input (Additional file 2: Table S2B2).

### *Prospective reliability study*

We tested revised knowledge and engagement scales for reliability (test-retest and internal scale consistency) in May 2016 among a convenience sample of stakeholders from the ongoing SEA (Sustainable Eating and Activity) Change and GenR8 Change community-based childhood obesity prevention initiatives in Victoria, Australia [35]. We used the same procedures as Phase 2, but with two-week test-retest reliability. No incentive was offered to participants. Procedures for individuals participating in research were approved by the Deakin University Human Ethics Advisory Group.

## Results

### Sample characteristics

We identified 13 coalition members from the SEA Change and GenR8 Change initiatives and acquired contact information for all members. All members agreed to participate in the reliability study, with 13 paired responses. The majority of the sample was female (*n* = 11; 84.6%) and had a Bachelor’s degree or higher (*n* = 11; 84.6%). The mean sample age was 41.8 years (SD = 12.0).

### Knowledge and engagement scores

Mean composite and domain-specific scores are reported in Table 3. On average, respondents agreed or strongly agreed with knowledge and engagement items.

**Table 3:** Phase 3 reliability results (*n* = 13 paired responses; SEA Change and GenR8 Change coalition members)

Construct and domains	# items	Max. score	Mean score (SD)^a^	Two-week test-retest reliability		Internal scale consistency (Cronbach’s α)		
				ICC (95% CI)	WSCV (95% CI)	*Test*	*Retest*	Average
Knowledge								
Composite	18	25	22.24 (1.28)	0.84 (0.62–0.95)	0.02 (0.01–0.03)	0.83	0.81	0.81
Domain-specific								
1. Problem	3	5	4.74 (0.43)	0.43 (0.11–0.82)	0.06 (0.04–0.09)	0.95	0.23	0.68
2. Intervention factors	6	5	4.60 (0.30)	0.67 (0.35–0.89)	0.03 (0.02–0.05)	0.72	0.44^b^	0.58
3. Roles	3	5	4.64 (0.35)	0.82 (0.57–0.94)	0.03 (0.02–0.05)	0.14	0.58	0.35
4. Sustainability	3	5	4.00 (0.51)	0.59 (0.25–0.86)	0.08 (0.05–0.11)	0.76	0.89	0.82
5. Resources	3	5	4.26 (0.58)	0.78 (0.51–0.92)	0.06 (0.04–0.09)	0.78	0.68	0.74
Engagement								
Composite	25	25	21.34 (1.77)	0.58 (0.23–0.86)	0.05 (0.03–0.08)	0.88	0.94	0.91
Domain-specific								
1. Dialogue & mutual learning	7	5	4.73 (0.32)	0.54 (0.20–0.85)	0.05 (0.04–0.08)	0.79	0.93	0.88
2. Flexibility	3	5	4.28 (0.56)	0.40 (0.09–0.82)	0.09 (0.06–0.14)	0.85	0.77	0.82
3. Influence & power	2	5	3.81 (0.83)	0.55 (0.21–0.85)	0.13 (0.09–0.21)	0.93	0.66	0.84
4. Leadership & stewardship	10	5	4.32 (0.43)	0.53 (0.19–0.84)	0.06 (0.04–0.09)	0.81	0.80	0.81
5. Trust	3	5	4.21 (0.32)	0.25 (0.02–0.84)	0.10 (0.07–0.15)	0.56	0.84	0.78

*ICC* intraclass correlation coefficient, *WSCV* within-subject coefficient of variation, *CI* confidence interval

^a^Scores calculated from *test* data. All items were on a 5-point agree/disagree Likert scale. Data were weighted to reflect the number of items per domain to ease domain-to-domain comparisons. Composite scores are a mean of the total, not a sum of means; therefore, domain scores may not add up to composite score

^b^One item was dropped in the analysis due to zero variance (“Preventing obesity early in life is important”)

### Knowledge and engagement reliability

Composite and domain-specific results are shown in Table 3, while per-item results are available in Additional file 4: Tables S3C1-C2. The ICCs for composite knowledge and engagement were 0.84 (95% CI: 0.62–0.95) and 0.58 (95% CI: 0.23–0.86), respectively. Corresponding WSCVs were 0.02 (95% CI: 0.01–0.03) and 0.05 (95% CI: 0.03–0.08). Across test-retest surveys, the average Cronbach’s α for composite knowledge and engagement internal scale consistencies were 0.81 and 0.91, respectively.

## Discussion

We developed and pilot-tested a novel survey that quantifies three potentially key properties of stakeholders involved in whole-of-community childhood obesity prevention interventions: social networks, knowledge, and engagement. In the retrospective study with stakeholders from SUS and R&C, we observed pre-post increases in recalled total mean knowledge scores, partly driven by increased understanding of modifiable factors to intervene on (SUS only) and available resources (R&C only). Stakeholders’ mean composite engagement scores did not change during the interventions but remained high. Domain-level change in engagement was only observed in SUS with a pre-post increase in stakeholders’ recalled influence and power. In this paper, we demonstrate the type of network data collected by the survey. Future prospective survey applications with further social network analysis will allow us to determine if patterns of connectivity among stakeholder groups exist, if there are changes and stability in group structures, and to identify opportunities to create cohesive relationships among stakeholders.

Phase 2 retrospective ICC values suggest excellent test-retest reliability for knowledge (ICC = 0.88) and engagement (ICC = 0.97) survey components [36]. We assessed reliability using T1 data because we assumed that participants could better recall their position at the start versus the end of intervention involvement.

The Phase 3 revised prospective survey represents how we intend to use the survey with ongoing interventions. The ICCs for composite knowledge and engagement scores were 0.84 and 0.58, which suggest excellent and fair-to-good test-retest reliability, respectively [36]. The decrease in engagement ICC from Phase 2 to Phase 3 may attribute to assessing test-retest reliability at one versus two weeks. The Phase 3 two-week assessment reflected concern of participant burden in repeating measurements in short turnaround times. We also present an alternative measure of test-retest reliability: within-subject coefficient of variation (WSCV). Both knowledge and engagement WSCVs were low (0.02 and 0.05, respectively), which indicates 2 and 5% variation in scores among test-retest participants. These findings increase our confidence in the survey’s test-retest reliability; however, further testing is needed to better understand construct dynamics over time.

Phase 3 knowledge and engagement scores were high on average, indicating that respondents agreed or strongly agreed with most survey items. Among this cross-sectional sample, respondents may have strongly understood and were invested in their communities’ childhood obesity prevention efforts. It is also possible that the survey needs further adaptations for local context and to capture greater variability in responses.

To our knowledge, the COMPACT Stakeholder-driven Community Diffusion Survey is the first survey developed that aims to examine change in social network, knowledge, and engagement properties of stakeholders involved in designing and implementing whole-of-community prevention interventions. Also using the CBPR Model as a guiding framework [12, 17], Zoellner and colleagues recently developed an instrument to assess community capacity of advisory board members planning a childhood obesity treatment program. While social networks were not assessed, the instrument included dimensions related to our knowledge (group roles, resources, sustainability) and engagement (communication, trust, participation and influence, leadership) domains [37]. The authors did not assess test-retest reliability but report good internal scale consistency for most dimensions (α > 0.7), similar to our Phase 3 scale reliability for knowledge (α = 0.8) and engagement (α = 0.9).

Our study strengths include an initial pilot test of the survey’s sensitivity among stakeholders involved in two whole-of-community interventions, SUS and R&C: studies that occurred nearly concurrently but far apart and with no communication between their stakeholders. The similar results across studies increases our confidence in applying the survey to diverse whole-of-community interventions in multiple geographies. Further, we included two rounds of reliability testing, which helped us modify the retrospective survey for prospective use.

Study limitations include small sample sizes and incomplete representation from SUS and R&C stakeholders due to nonresponse and inability to acquire contact information. Phase 2 data were collected retrospectively and responses may be inaccurate due to recall and memory issues. We did not assess the reliability of the social network survey module; however, our type of name generator questions have been extensively used across varied survey research settings [25], and we followed best practices in guarding against recall biases in formulating our research design and question wording [38, 39].

To increase our understanding of how Stakeholder-driven Community Diffusion operates within whole-of-community interventions, future work will use insights from system science [40–42]. One approach is agent-based modeling, which simulates individuals interacting with one another and their environment with specified behavioral rules [43]. We will use SUS and R&C data from this study to parameterize, calibrate, and test models that demonstrate knowledge and engagement diffusion throughout social networks.

Additionally, we are currently using the COMPACT Stakeholder-driven Community Diffusion Survey prospectively to evaluate early childhood obesity prevention studies in Somerville, Massachusetts, USA and Auckland, New Zealand. By having data from multiple interventions in communities across the world, we aim to iteratively develop and rigorously test an agent-based model with wide applicability. Social network, knowledge, and engagement data from the survey may also be used to inform community intervention efforts in real-time (e.g., to convene stakeholders with high connectivity to others; to implement stakeholder leadership trainings; to develop community-wide channels for disseminating information and available resources related to obesity prevention). We expect to further adapt the survey based on longitudinal study insights and participant feedback. Future research is needed to identify potential sources of response error and to assess the reliability and validity of revised surveys among larger samples, including predictive validity for implementation outcomes.

## Conclusion

Whole-of-community interventions may be a major potential response to curbing the childhood obesity epidemic. Tailoring precise prevention interventions to community characteristics and contexts, for example stakeholders’ social network structures, knowledge, and engagement, may lead to sustained success [18]. If that is true, then the novel survey developed and evaluated for this paper could be a key piece of that tailoring.

## Additional files

Additional file 1: **Table S1A**. Engagement literature review and scale development. (DOCX 54 kb)

Additional file 2: **Table S2B1-B2**. Phase 2 retrospective Shape Up Somerville per-item knowledge and engagement reliability results (*n* = 11 paired responses). Data from test-retest surveys administered online one week apart in May and June 2015: members of the 2003–2005 Shape Up Somerville Community Advisory Council. (DOCX 33 kb)

Additional file 3: **Appendix**. Example COMPACT Stakeholder-driven Community Diffusion Survey. (DOCX 39 kb)

Additional file 4: **Table S3C1-C2**. Phase 3 prospective per-item knowledge and engagement reliability results (*n* = 13 paired responses). Data from test-retest surveys administered online two weeks apart in May 2016: members of the SEA Change and GenR8 Change coalitions in Victoria, Australia. (DOCX 28 kb)

## Abbreviations

CBPR: Community-based participatory research
CI: Confidence interval
COMPACT: Childhood Obesity Modeling for Prevention And Community Transformation
ICC: Intraclass correlation coefficient
R&C: Romp & Chomp
SD: Standard deviation
SUS: Shape Up Somerville
WSCV: Within-subject coefficient of variation
