# Supplementary material for: Development and testing of a novel survey to assess Stakeholder-driven Community Diffusion of childhood obesity prevention efforts
Source: BMC Public Health. 2018 May 31;18:681. doi: 10.1186/s12889-018-5588-1 (PMC5984309; doi:10.1186/s12889-018-5588-1)
Supplement: Supplementary file 1 — Table S1A. Engagement literature review and scale development. (DOCX 54 kb) [file 12889_2018_5588_MOESM1_ESM.docx]

**Table A. Engagement literature review and scale development**

| **Item source** | **Cited in CBPR matrix?** [1] | **Original item** | **Adapted item for Phase 2 SUS retrospective scale** | **Expert score**  **(out of 12)**^a^ |  |
| --- | --- | --- | --- | --- | --- |
| **Domain 1: Dialogue & mutual learning** | | | | | |
| Community coalitions: a self-assessment tool [2] | Yes | Members are regularly involved in meetings and/or activities | I was regularly involved in SUS intervention meetings and/or activities | 10 |  |
| Instrument for evaluating dimensions of group dynamics within CBPR partnerships [3] | Yes | How often do you suggest new ideas? | I frequently suggested new ideas | 9 |  |
| Team Performance Survey [4] | No | Team members listened to each other when someone expressed a concern about individual or team performance | I listened to other CAC members when someone expressed a concern | 9 |  |
| Team Performance Survey [4] | No | All team members made an effort to participate in discussions | I made an effort to participate in discussions | 9 |  |
| Team Assessment Questionnaire [5] | No | I share my ideas/suggestions whether or not my boss agrees with my input | I shared my ideas/suggestions whether or not CAC members agreed with my input | 9 |  |
| Team Assessment Questionnaire [5] | No | Team members can openly discuss their own problems and issues | I could openly discuss problems and issues | 8 |  |
| Team Performance Survey [4] | No | Team members seemed attentive to what other team members were saying when they spoke | I was attentive to what other CAC members were saying when they spoke | 8 |  |
| Team Diagnostic Survey [6] | No | The team leader works with the team to develop the best possible approach to its work | I worked with the CAC to develop the best possible approach to our work | 8 |  |
| Coalition Self-Assessment Survey [7] | Yes | I can talk openly and honestly at the coalition meetings | I could talk openly and honestly at CAC meetings | 7 |  |
| Internal Coalition Effectiveness [8] | Yes | Leaders facilitate a shared social vision among coalition members | I shared a social vision among CAC members | 7 |  |
| Wilder Collaboration Factors Inventory [9] | Yes | My ideas about what we want to accomplish with this collaboration seem to be the same as the ideas of others | My ideas about what CAC wanted to accomplish seemed to be the same as the ideas of others | 7 |  |
| **Domain 2: Flexibility** | | | | | |
| Wilder Collaboration Factors Inventory [9] | Yes | This collaboration is able to adapt to changing conditions, such as fewer funds than expected, or changing political climate, or change in leadership | I was able to adapt to changing conditions, such as fewer funds than expected, or change in political climate, or change in leadership | 11 |  |
| Wilder Collaboration Factors Inventory [9] | Yes | People in this collaborative group are open to different approaches to how we can do our work. They are willing to consider different ways of working. | I was open to different approaches to how the CAC did its work | 10 |  |
| Wilder Collaboration Factors Inventory [9] | Yes | People in our collaboration are willing to compromise on important aspects of our project | I was willing to compromise on important aspects of the SUS intervention | 9 |  |
| Building International Collaborative Capacity [10] | No | We included the interests and ideas of all members in the job plan | I included the interests and ideas of all CAC members | 8 |  |
| Team Performance Survey [4] | No | Different points of view were respected by team members | I respected different points of view from CAC members | 8 |  |
| Wilder Collaboration Factors Inventory [9] | Yes | People in this collaborative group are open to different approaches to how we can do our work. They are willing to consider different ways of working. | I was willing to consider different ways of working | 8 |  |
| Team Performance Survey [4] | No | Team members worked to come up with solutions that satisfied all members | I worked to come up with solutions that satisfied all CAC members | 8 |  |
| Team Performance Survey [4] | No | My team actively elicited multiple points of view before deciding on a final answer | I actively elicited multiple points of view before deciding on a final answer | 6 |  |
| **Domain 3: Influence & power (4 items)** | | | | | |
| Transformational Leadership in Work Groups [11] | Yes | I have a great deal of control over what happens in my department | I had a great deal of control over what happened in the CAC | 9 |  |
| Health Education and Community Empowerment [12] | Yes | I can influence decisions that affect my community | I influenced decisions that affected the SUS intervention | 9 |  |
| Community Partnership Member Survey [13] | Yes | How much influence do you have in determining the policies and actions of the committee? | I influenced the policies and actions of the SUS intervention | 9 |  |
| Health Education and Community Empowerment [12] | Yes | I can influence the decisions that this organization makes | I influenced the decisions that the CAC made | 7 |  |
| **Domain 4: Leadership & stewardship** | | | | | |
| Internal Coalition Effectiveness [8] | Yes | Leaders establish positive relationships and strong links with community members that the coalition wants to engage and mobilize | I established positive relationships with community members that the CAC wanted to engage and mobilize | 10 |  |
| Team Assessment Questionnaire [5] | No | My boss leads by example | I lead by example | 10 |  |
| Coalition Self-Assessment Survey [7] | Yes | Coalition members take responsibility for getting the work done | I took responsibility for getting the work done | 10 |  |
| Wilder Collaboration Factors Inventory [9] | Yes | The people in this collaborative group are dedicated to the idea that we can make this project work | I was dedicated to the idea that the CAC could make the SUS intervention work | 10 |  |
| Communication for Social Change [14] | Yes | How would you describe each leader in the issue/program's enthusiasm/passion for strengthening/motivating community aspirations for the issue/program | My passion and enthusiasm for SUS motivated community aspiration for the project | 10 |  |
| Team Performance Survey [4] | No | Team members encouraged one another to express their opinions and thoughts | I encouraged CAC members to express their opinions and thoughts | 9 |  |
| Team Diagnostic Survey [6] | No | The team leader helps the team build a high shared commitment to its purposes | I helped the CAC build a high shared commitment to its purposes | 9 |  |
| Team Diagnostic Survey [6] | No | The team leader helps the team sustain the motivation of all members | I helped the CAC sustain the motivation of all members | 9 |  |
| Coalition Effectiveness Inventory [13] | Yes | Leaders provide leadership and guidance in maintaining coalition | I provided leadership and guidance in maintaining the CAC | 9 |  |
| Team Diagnostic Survey [6] | No | Regular team members take initiatives to help the team build and use well members' knowledge and skills | I took initiative to help the team build and use well CAC members' knowledge and skills | 9 |  |
| Team Diagnostic Survey [6] | No | Everyone on this team is motivated to have the team succeed | I was motivated to have the SUS intervention succeed | 9 |  |
| Coalition Self-Assessment Survey [7] | Yes | The leadership of our coalition advocates strongly for its own opinions and agendas | I advocated strongly for my own opinions and agendas | 8 |  |
| Coalition Self-Assessment Survey [7] | Yes | The leadership of our coalition builds consensus on key decisions | I built consensus on key decisions | 8 |  |
| Team Assessment Questionnaire [5] | No | My boss coaches and supports individual team members | I coached and supported individual CAC members | 8 |  |
| Development of Scales Measuring the Capacity of Community-Based Initiatives [15] | No | People involved in the project do not give up when the project faces challenges | I did not give up when the CAC faced challenges | 8 |  |
| Transformational Leadership in Work Groups [11] | Yes | My team leader emphasizes the importance of having a collective sense of mission | I emphasized the importance of having a collective sense of mission | 8 |  |
| Wilder Collaboration Factors Inventory [9] | Yes | The people in leadership positions for this collaboration have good skills for working with other people and organizations | I had good skills for working with other people and organizations | 8 |  |
| Team Performance Survey [4] | No | Team members resolved differences of opinion by openly speaking their mind | I helped resolve differences of opinion | 8 |  |
| Team Diagnostic Survey [6] | No | Regular team members take initiatives to constructively resolve any problems or conflicts that develop among members | I took initiative to constructively resolve any problems or conflicts that developed among the CAC | 8 |  |
| Team Diagnostic Survey [6] | No | Regular team members take initiatives to promote high shared motivation and commitment | I took initiative to promote high shared motivation | 8 |  |
| Development of Scales Measuring the Capacity of Community-Based Initiatives [15] | No | The leadership tries to develop agreement in group decision making | I tried to develop agreement in group decision making | 8 |  |
| Development of Scales Measuring the Capacity of Community-Based Initiatives [15] | No | The leadership's vision is clear to people involved with the project | My vision was clear to people involved with the SUS intervention | 7 |  |
| **Domain 5: Trust (5 items)** | | | | | |
| Interpersonal Collaboration Scale [16] | No | The members of our center have a high level of mutual trust in each other | CAC members had a high level of trust in me | 10 |  |
| Collaborative Productivity Scale [17] | No | In general, I feel that I can trust the colleagues with whom I collaborate | In general, I felt that I could trust CAC members with whom I collaborated | 10 |  |
| Wilder Collaboration Factors Inventory [9] | Yes | People involved in our collaboration always trust one another | People involved in CAC trusted me | 9 |  |
| Interpersonal Collaboration Scale [16] | No | The PI/Director/Leader of our center is effective in promoting a climate of collaboration and trust | I tried to promote a climate of collaboration and trust | 8 |  |
| Team Assessment Questionnaire [5] | No | Team members trust each other | I trusted other CAC members | 8 |  |

*Notes.* SUS = Shape Up Somerville; CAC = Community Advisory Council.

^a^Six experts scored 104 items from existing instruments to evaluate content validity (0=no; 1=maybe; 2=yes). We eliminated items with low scores (less than 6 points) or if deemed duplicative, yielding 50 items in the Phase 2 retrospective engagement scale. All items were adapted to fit a 5-point agree/disagree Likert scale.

**References**

1. Sandoval JA, Lucero J, Oetzel J, Avila M, Belone L, Mau M, Pearson C, Tafoya G, Duran B, Iglesias Rios L *et al*: Process and outcome constructs for evaluating community-based participatory research projects: a matrix of existing measures. *Health education research* 2012, 27(4):680-690.

2. Goldstein SM: Community coalitions: a self-assessment tool. *Am J Health Promot* 1997, 11(6):430-435.

3. Schulz AJ, Israel BA, Lantz P: Instrument for evaluating dimensions of group dynamics within community-based participatory research partnerships. *Evaluation and Program Planning* 2003, 26(3):249-262.

4. Thompson BM, Levine RE, Kennedy F, Naik AD, Foldes CA, Coverdale JH, Kelly PA, Parmelee D, Richards BF, Haidet P: Evaluating the quality of learning-team processes in medical education: development and validation of a new measure. *Academic medicine : journal of the Association of American Medical Colleges* 2009, 84(10 Suppl):S124-127.

5. Department of Defense, Agency for Healthcare Research and Quality. Team Assessment Questionnaire. <https://www.teamsciencetoolkit.cancer.gov/public/TSResourceMeasure.aspx?tid=2&rid=443>. Accessed 31 October 2017.

6. Wageman R, Hackman JR, Lehman E: Team Diagnostic Survey: Development of an Instrument. *The Journal of Applied Behavioral Science* 2005, 41(4):373-398.

7. Kenney E, Sofaer S: The Coalition Self Assessment Survey: A manual for users. *New York: School of Public Affairs, Baruch College* 2000.

8. Cramer ME, Atwood JR, Stoner JA: Measuring community coalition effectiveness using the ICE instrument. *Public health nursing (Boston, Mass)* 2006, 23(1):74-87.

9. Mattessich P, Murray-Close, M., Monsey, B.: Wilder Collaboration Factors Inventory. In. St. Paul, MN: Amherst H. Wilder Foundation; 2001.

10. Garcia-Ramirez M, Paloma V, Suarez-Balcazar Y, Balcazar F: Building international collaborative capacity: contributions of community psychologists to a European network. *American journal of community psychology* 2009, 44(1-2):116-122.

11. Jung DI, Sosik JJ: Transformational Leadership in Work Groups: The Role of Empowerment, Cohesiveness, and Collective-Efficacy on Perceived Group Performance. *Small Group Research* 2002, 33(3):313-336.

12. Israel BA, Checkoway B, Schulz A, Zimmerman M: Health education and community empowerment: conceptualizing and measuring perceptions of individual, organizational, and community control. *Health education quarterly* 1994, 21(2):149-170.

13. Butterfoss FD, Goodman RM, Wandersman A: Community coalitions for prevention and health promotion: factors predicting satisfaction, participation, and planning. *Health education quarterly* 1996, 23(1):65-79.

14. Maria Elena Figueroa, D. Lawrence Kincaid, Manju Rani, Gary Lewis: Communication for Social Change: An Integrated Model for Measuring the Process and Its Outcomes. In. New York, New York 2002.

15. Lempa M, Goodman RM, Rice J, Becker AB: Development of scales measuring the capacity of community-based initiatives. *Health education & behavior : the official publication of the Society for Public Health Education* 2008, 35(3):298-315.

16. National Cancer Institute Science of Team Science (SciTS) Team. Interpersonal Collaboration Scale. <https://www.teamsciencetoolkit.cancer.gov/public/TSResourceMeasure.aspx?tid=2&rid=391>. Accessed 31 October 2017.

17. Hall KL, Stokols D, Moser RP, Taylor BK, Thornquist MD, Nebeling LC, Ehret CC, Barnett MJ, McTiernan A, Berger NA *et al*: The collaboration readiness of transdisciplinary research teams and centers findings from the National Cancer Institute's TREC Year-One evaluation study. *American journal of preventive medicine* 2008, 35(2 Suppl):S161-172.
