# Supplementary material for: Development and testing of a novel survey to assess Stakeholder-driven Community Diffusion of childhood obesity prevention efforts
Source: BMC Public Health. 2018 May 31;18:681. doi: 10.1186/s12889-018-5588-1 (PMC5984309; doi:10.1186/s12889-018-5588-1)
Supplement: Supplementary file 2 — Table S2B1-B2. Phase 2 retrospective Shape Up Somerville per-item knowledge and engagement reliability results (n = 11 paired responses). Data from test-retest surveys administered online one week apart in May and June 2015: members of the 2003–2005 Shape Up Somerville Community Advisory Council. (DOCX 33 kb) [file 12889_2018_5588_MOESM2_ESM.docx]

**Table B1. Phase 2 retrospective Shape Up Somerville per-item knowledge reliability results (n=11 paired responses). Data from test-retest surveys administered online one week apart in May and June 2015: members of the 2003-05 Shape Up Somerville Community Advisory Council^a^**

| **#** | **Item**^b^ | **Weighted Kappa (κ_w_)** |  |
| --- | --- | --- | --- |
| **Domain 1: The problem of childhood obesity (Problem; 4 items)** | | | |
| 1 | Childhood obesity is a problem in Somerville a) True; b) False; c) Not sure | 0.23 |  |
| 2 | What is the most cost-effective approach for childhood obesity? a) Treatment; b) Prevention; c) Not sure | 1.00 |  |
| 3 | Childhood obesity is associated with increased risk of… a) Food allergies; b) Low blood pressure; c) Type II diabetes; d) Not sure | -0.06 |  |
| 4 | Which of the following decreases a child's risk of obesity? a) Allowing a child to eat when they are hungry and stop when they are full; b) Having an overweight or obese parent; c) Sugar-sweetened beverage consumption; d) Having a TV where a child sleeps; e) Not sure | 0.30 |  |
| **Domain 2: Modifiable determinants of childhood obesity and level of social ecology to address them** **(Intervention factors; 4 items)** | | | |
| 5 | On a population level, which strategy is most effective in preventing childhood obesity? a) Changing policies and the environment; b) Educational interventions; c) Changing individual habits; d) Not sure | 0.55 |  |
| 6 | Which intervention would have the greatest reach and impact in increasing children's physical activity? a) Encouraging parents to promote physical activity at home; b) Increasing active play time in school; c) Increasing community access to safe parks and recreational space; d) Not sure | 0.44 |  |
| 7 | Which determinant is the least important to address in community-based obesity prevention? a) Screen time; b) Diet; c) Physical activity; d) Genetics; e) Not sure | 0.57 |  |
| 8 | Certain populations, including racial/ethnic minorities and those of lower socioeconomic status, are more vulnerable to obesity a) True; b) False; c) Not sure | -0.06 |  |
| **Domain 3: Stakeholders’ roles in the whole intervention, what others are doing, and knowledge of multi-setting components (Roles; 4 items)** | | | |
| 9 | Stakeholders involved in a childhood obesity intervention should: a) Understand what role they play; b) Understand what others are doing in the intervention; c) Both a and b; d) None of the above; e) Not sure | 0.39 |  |
| 10 | Which of the following is most important for successful community-based childhood obesity interventions? a) Collaboration among people within the same setting; b) Collaboration with people across different settings; c) Working in isolation; d) Not sure | 0.56 |  |
| 11 | To have the greatest community level impact, where should childhood obesity interventions focus their efforts? a) Schools; b) Pediatric offices; c) After-school programs; d) All of the above; e) Not sure | 0.48 |  |
| 12 | When working with a community, a multi-setting childhood obesity intervention is more effective than a single-setting approach a) True; b) False; c) Not sure | 1.00 |  |
| **Domain 4: How to intervene to achieve sustainability (Sustainability; 4 items)** | | | |
| 13 | When is the most optimal time to start preventing childhood obesity? a) During early childhood; b) During elementary school; c) During adolescence; d) Not sure | 0.32 |  |
| 14 | Which of the following intervention components, at a community level, is least effective in promoting healthy child weight? a) Reducing unhealthy food marketing in public sites; b) Providing professional training for primary care providers to encourage healthy eating; c) A one-time promotional campaign to limit screen time; d) Not sure | 0.62 |  |
| 15 | Which intervention component does not contribute to the sustainability of a childhood obesity intervention? a) Implementation of a new school wellness policy; b) Dissemination of nutrition and physical activity curricula across the state; c) Sending newsletters home with parents; d) Not sure | -0.12 |  |
| 16 | When should the sustainability of a childhood obesity intervention be considered? a) During the planning phase; b) During the implementation phase; c) When funding is running low; d) Not sure | 0.62 |  |
| **Domain 5: Available resources (Resources; 4 items)** | | | |
| 17 | I invested my own resources so that adequate SUS related policies and plans were developed for the whole community  (4-point agree/disagree Likert scale) | -0.03 |  |
| 18 | I invested in education and training of Community Advisory Council members to facilitate the achievement of our objectives  (4-point agree/disagree Likert scale) | 0.00 |  |
| 19 | I invested in developing and maintaining social relations between members of the Community Advisory Council  (4-point agree/disagree Likert scale) | 0.15 |  |
| 20 | I was aware of any evaluation efforts in place to address childhood obesity (4-point agree/disagree Likert scale) | 0.28 |  |

*Notes.* SUS = Shape Up Somerville.

^a^Reliability results from T1, i.e., the start of Community Advisory Council members’ involvement in the SUS intervention.

^b^Correct responses for multiple choice and true/false items are underlined.

**Table B2. Phase 2 retrospective Shape Up Somerville per-item engagement reliability results (n=11 paired responses). Data from test-retest surveys administered online one week apart in May and June 2015: members of the 2003-05 Shape Up Somerville Community Advisory Council^a^**

| **#** | **Item** | **Weighted Kappa (κ_w_)** | **Item kept for Phase 3 prospective tool?** | **Reason for exclusion** |  |
| --- | --- | --- | --- | --- | --- |
| **Domain 1: Dialogue & mutual learning (11 items)** | | | | | |
| 1 | I was regularly involved in SUS intervention meetings and/or activities | 0.25 | N | Low kappa |  |
| 2 | I made an effort to participate in discussions | 0.72 | Y | - |  |
| 3 | I listened to other CAC members when someone expressed a concern | 0.40 | Y | - |  |
| 4 | I was attentive to what other CAC members were saying when they spoke | 0.47 | Y | - |  |
| 5 | I frequently suggested new ideas | 0.49 | N | Low T2 kappa  (data not shown) |  |
| 6 | I shared my ideas/suggestions whether or not CAC members agreed with my input | 0.56 | Y | - |  |
| 7 | I could openly discuss problems and issues | 0.66 | Y | - |  |
| 8 | I could talk openly and honestly at CAC meetings | 0.57 | Y | - |  |
| 9 | My ideas about what the CAC wanted to accomplish seemed to be the same as the ideas of others | 0.51 | N | Low T2 kappa  (data not shown) |  |
| 10 | I shared a social vision among CAC members | 0.12 | N | Low kappa |  |
| 11 | I worked with the CAC to develop the best possible approach to our work | 0.57 | Y | - |  |
| **Domain 2: Flexibility (8 items)** | | | | | |
| 12 | I was able to adapt to changing conditions, such as fewer funds than expected, or change in political climate, or change in leadership | 0.27 | N | Low kappa |  |
| 13 | I was open to different approaches to how the CAC did its work | 0.13 | N | Low kappa |  |
| 14 | I was willing to consider different ways of working | 0.20 | N | Low kappa |  |
| 15 | I was willing to compromise on important aspects of the SUS intervention | 0.51 | Y | - |  |
| 16 | I included the interests and ideas of all CAC members | 0.47 | N | Low T2 kappa  (data not shown) |  |
| 17 | I worked to come up with solutions that satisfied all CAC members | 0.45 | Y | - |  |
| 18 | I respected different points of view from CAC members | 0.39 | Y | - |  |
| 19 | I actively elicited multiple points of view before deciding on a final answer | 0.29 | N | Low kappa |  |
| **Domain 3: Influence & power (4 items)** | | | | | |
| 20 | I had a great deal of control over what happened in the CAC | 0.00 | N | Low T2 kappa  (data not shown) |  |
| 21 | I influenced the decisions that the CAC made | 0.20 | N | Low kappa |  |
| 22 | I influenced decisions that affected the SUS intervention | 0.49 | Y | - |  |
| 23 | I influenced the policies and actions of the SUS intervention | 0.38 | Y | - |  |
| **Domain 4: Leadership & stewardship (22 items)** | | | | | |
| 24 | I was dedicated to the idea that the CAC could make the SUS intervention work | 0.39 | N | Redundant with #25 |  |
| 25 | I was motivated to have the SUS intervention succeed | 0.62 | Y | - |  |
| 26 | My passion and enthusiasm for the SUS intervention motivated community aspiration for the project | 0.56 | Y | - |  |
| 27 | I took responsibility for getting the work done | -0.02 | N | Low kappa |  |
| 28 | I established positive relationships with community members that the CAC wanted to engage and mobilize | 0.60 | Y | - |  |
| 29 | I had good skills for working with other people and organizations | 0.76 | Y | - |  |
| 30 | I led by example | 0.49 | Y | - |  |
| 31 | I encouraged CAC members to express their opinions and thoughts | 0.57 | Y | - |  |
| 32 | I helped the CAC build a high shared commitment to its purposes | 0.47 | N | Confusing wording |  |
| 33 | I emphasized the importance of having a collective sense of mission | 0.47 | Y | - |  |
| 34 | I provided leadership and guidance in maintaining the CAC | 0.32 | Y | - |  |
| 35 | I coached and supported individual CAC members | 0.05 | N | Low kappa |  |
| 36 | I helped the CAC sustain the motivation of all members | 0.15 | N | Low kappa |  |
| 37 | I took initiative to promote high shared motivation | 0.17 | N | Low kappa |  |
| 38 | I took initiative to help the CAC build and use well members' knowledge and skills | 0.19 | N | Low kappa |  |
| 39 | I helped resolve differences of opinion | -0.08 | N | Low kappa |  |
| 40 | I took initiative to constructively resolve any problems or conflicts that developed among the CAC | 0.11 | N | Low kappa |  |
| 41 | I advocated strongly for my own opinions and agendas | 0.58 | Y | - |  |
| 42 | I built consensus on key decisions | 0.30 | N | Low T2 kappa  (data not shown) |  |
| 43 | I tried to develop agreement in group decision making | 0.15 | N | Low kappa |  |
| 44 | I did not give up when CAC faced challenges | 0.33 | Y | - |  |
| 45 | My vision was clear to people involved with the SUS intervention | 0.20 | N | Low kappa |  |
| **Domain 5: Trust (5 items)** | | | | | |
| 46 | In general, I felt that I could trust CAC members with whom I collaborated | 0.45 | N | Low T2 kappa  (data not shown) |  |
| 47 | I trusted other CAC members | 0.57 | Y | - |  |
| 48 | CAC members had a high level of trust in me | 0.60 | N | Redundant with #49 |  |
| 49 | People involved in the CAC trusted me | 0.64 | Y | - |  |
| 50 | I tried to promote a climate of collaboration and trust | 0.71 | Y | - |  |

*Notes.* SUS = Shape Up Somerville; CAC = Community Advisory Council; Y = yes; N = no.

^a^Reliability results from T1, i.e., the start of Community Advisory Council members’ involvement in the SUS intervention. T2 = end of CAC members’ involvement in the SUS intervention (data not shown)).
