# Supplementary material for: Development and testing of a novel survey to assess Stakeholder-driven Community Diffusion of childhood obesity prevention efforts
Source: BMC Public Health. 2018 May 31;18:681. doi: 10.1186/s12889-018-5588-1 (PMC5984309; doi:10.1186/s12889-018-5588-1)
Supplement: Supplementary file 3 — Appendix. Example COMPACT Stakeholder-driven Community Diffusion Survey. (DOCX 39 kb) [file 12889_2018_5588_MOESM3_ESM.docx]

**Appendix. Example COMPACT Stakeholder-driven Community Diffusion Survey**

**Overview:** The COMPACT Stakeholder-driven Community Diffusion Survey captures quantitative, longitudinal information from community stakeholders (e.g., members of steering committees, coalitions, or community advisory boards) on levels of knowledge and engagement and to identify social relationships related to whole-of-community childhood obesity prevention efforts. The survey also includes a brief demographic questionnaire. The survey has been used both retrospectively and prospectively in Somerville, Massachusetts, USA; Greenville, South Carolina, USA; Victoria, Australia; Auckland, New Zealand and is being refined and adapted.

**Example survey used in the Shape Up Under 5 study (Somerville, Massachusetts):**

*Target respondents:* Members of the Shape Up Under 5 Committee (primary); committee members’ social network ties related to childhood obesity prevention efforts (secondary).

*Frequency and timing of administration:* Baseline, 6, 12, 18, 24 months. Note: committee social network data were collected every 3 months and demographics were only collected at baseline.

*Mode of administration:* Self-administered online using Qualtrics.

**Development and conditions of use:** The survey was developed by members of the COMPACT study team in 2014-15. This work was supported by the National Institutes of Health (NHLBI and OBSSR, R01HL115485) and the Brookings Institution.

For use and/or adaptations of this instrument, please contact:

Christina Economos, Ph.D.

Professor

New Balance Chair in Childhood Nutrition

Gerald J. and Dorothy R. Friedman School of Nutrition Science and Policy

Tufts University

150 Harrison Ave.
Boston, MA 02111

Phone: 617-636-3784

Email: [Christina.Economos@tufts.edu](mailto:Christina.Economos@tufts.edu)

**INSTRUCTIONS** [*note*: text in gray represent instructions for survey administrator]

Thank you for your participation in the Shape Up Under 5 study. This survey will take approximately 15 minutes of your time. If you’d like to take a break from the survey, your responses will automatically be saved when you exit the page. To return to the survey, re-click the link found in your email and you will be brought to the question where you last left off. Please note that there is a bar at the top of the screen that shows your progress with the survey from 0 to 100%. All responses will be kept confidential, and only research staff will have access to the responses.

This survey includes questions on the following topics:

1. Your perspectives and understanding of early childhood* obesity prevention
2. Your engagement with the issue, which includes your involvement, adaptability, influence, leadership, and trustworthiness
3. Your professional relationships
4. Demographic information

*When we refer to **early childhood**, we mean children ages 0 to 5 years.

Please type in your name in the space below and then click “begin” to start the survey:

_______________________

[PAGE BREAK]

**Section 1 [KNOWLEDGE]**

The following questions will ask you about your perspectives and understanding of early childhood obesity prevention in Somerville.

To what extent do you agree or disagree with the following statements?

|  |  | Strongly disagree  (1) | Disagree  (2) | Neutral  (3) | Agree  (4) | Strongly agree  (5) |
| --- | --- | --- | --- | --- | --- | --- |
| **Domain 1: The problem of childhood obesity (Problem)** | | | | | | |
| 1 | Early childhood obesity is a problem in Somerville |  |  |  |  |  |
| 2 | There are increased health care costs due to medical complications of obesity in early childhood |  |  |  |  |  |
| 3 | I am familiar with other illnesses and health concerns associated with obesity in early childhood |  |  |  |  |  |
| **Domain 2: Modifiable determinants of childhood obesity and level of social ecology to address them (Intervention factors)** | | | | | | |
| 4 | Preventing obesity early in life is important |  |  |  |  |  |
| 5 | Certain populations of children are more vulnerable to obesity |  |  |  |  |  |
| 6 | I feel confident in listing risk factors related to early childhood obesity |  |  |  |  |  |
| 7 | I am aware of evidence-based strategies that target risk factors related to early childhood obesity |  |  |  |  |  |
| **Domain 4: How to intervene to achieve sustainability (Sustainability)** | | | | | | |
|  | I know strategies to prevent obesity in early childhood that… |  |  |  |  |  |
| 8 | Will be acceptable and appropriate for the Somerville community |  |  |  |  |  |
| 9 | Will have the greatest impact in promoting healthy weight |  |  |  |  |  |
| 10 | Can be sustained over time |  |  |  |  |  |
| **Domain 3: Stakeholders’ roles in the whole intervention, what others are doing, and knowledge of multi-setting components (Roles)** | | | | | | |
| 11 | I can play a role in preventing early childhood obesity in Somerville |  |  |  |  |  |
| 12 | I know what is being done in the Somerville community to prevent early childhood obesity |  |  |  |  |  |
| 13 | Obesity prevention strategies should be prioritized and implemented by multiple early childhood providers |  |  |  |  |  |
| **Domain 5: Available resources (Resources)** | | | | | | |
| 14 | I know where to find resources related to early childhood obesity prevention |  |  |  |  |  |
| 15 | I am aware of staff education or training opportunities that include the subject of early childhood obesity prevention |  |  |  |  |  |
| 16 | I am aware of evaluation and monitoring efforts in Somerville that address childhood obesity in young children |  |  |  |  |  |
| **Domain 1 (continued): What level to intervene upon and which modifiable determinants of childhood obesity to address (Intervention factors)** | | | | | | |
| 17 | I feel confident in listing possible policy changes to combat early childhood obesity in Somerville |  |  |  |  |  |
| 18 | I feel confident in suggesting changes in early childhood settings (like child care, medical settings, or play areas) to prevent early childhood obesity |  |  |  |  |  |

[PAGE BREAK]

**Section 2 [ENGAGEMENT]**

This section of the survey will ask you about your *engagement* with the topic of early childhood obesity prevention in Somerville. In this study, we refer to *engagement* as an individual’s sense of involvement, adaptability, influence, leadership, and trustworthiness. In addition, we use the term *colleagues* in this survey to mean coworkers (e.g., staff and supervisors/managers) and peers in the community (e.g., collaborators and fellow committee or coalition members).

To what extent do you agree or disagree with the following statements?

|  |  | Strongly disagree  (1) | Disagree  (2) | Neutral  (3) | Agree  (4) | Strongly agree  (5) |
| --- | --- | --- | --- | --- | --- | --- |
| **Domain 1: Dialogue & mutual learning** | | | | | | |
| 1 | I can talk openly and honestly at work or meetings |  |  |  |  |  |
| 2 | I make an effort to participate in discussions |  |  |  |  |  |
| 3 | I listen to colleagues when someone expresses a concern |  |  |  |  |  |
| 4 | I am attentive to what colleagues say when they speak |  |  |  |  |  |
| 5 | I share my ideas and suggestions whether or not colleagues agree with my input |  |  |  |  |  |
| 6 | I can openly discuss problems and issues |  |  |  |  |  |
| 7 | I work with colleagues to develop the best possible approach to our work |  |  |  |  |  |
| **Domain 2: Flexibility** | | | | | | |
| 8 | I am willing to make compromises related to my work in early childhood obesity prevention |  |  |  |  |  |
| 9 | I work to come up with solutions that satisfy all colleagues |  |  |  |  |  |
| 10 | I respect different points of view from colleagues |  |  |  |  |  |
| **Domain 3: Influence & power** | | | | | | |
| 11 | I influence decisions that affect early childhood obesity prevention efforts in the community |  |  |  |  |  |
| 12 | I influence the policies and actions of community-based early childhood obesity prevention |  |  |  |  |  |
| **Domain 4: Leadership & stewardship** | | | | | | |
| 13 | I am motivated to prevent early childhood obesity |  |  |  |  |  |
| 14 | My passion and enthusiasm for early childhood obesity prevention motivates others |  |  |  |  |  |
| 15 | I establish positive relationships with community members with whom my colleagues want to engage and mobilize |  |  |  |  |  |
| 16 | I have good skills for working with other people and organizations |  |  |  |  |  |
| 17 | I lead by example |  |  |  |  |  |
| 18 | I encourage colleagues to express their opinions and thoughts |  |  |  |  |  |
| 19 | I emphasize the importance of having a collective sense of mission |  |  |  |  |  |
| 20 | I provide leadership and guidance in maintaining relationships among colleagues |  |  |  |  |  |
| 21 | I advocate strongly for my own opinions and agendas |  |  |  |  |  |
| 22 | I do not give up when faced with challenges |  |  |  |  |  |
| **Domain 5: Trust** | | | | | | |
| 23 | I trust others involved in early childhood obesity prevention efforts |  |  |  |  |  |
| 24 | People involved in early childhood obesity prevention efforts trust me |  |  |  |  |  |
| 25 | I try to promote a climate of collaboration and trust |  |  |  |  |  |

[PAGE BREAK]

**Section 3 [SOCIAL NETWORKS]**

This section of the survey will ask you to identify people with whom you discuss issues related to early childhood obesity prevention in Somerville. All responses will be kept confidential, and only research staff will have access to the responses.

[PAGE BREAK]

1. Please provide the names of people with whom you discuss issues related to early childhood obesity in Somerville. You can mention up to 20 names and please enter the name of each person in its own entry box. The order of the names is not important. If you cannot remember a person’s full name, try to include as much descriptive information as you can.

To help you get started, these people may be associated with the following groups:

●University ●WIC ●Early education ●Center-based child care

●Home-based child care ●Healthcare ●Health Department

●Community-based organization ●City of Somerville ●Parent

[insert 20 blank fields]

[PAGE BREAK]

1. To the best of your knowledge, please select each person’s group affiliation from the drop down list. If applicable, try to also enter each person’s organization or department and title in the spaces provided. It is okay to leave their organization/department blank if you think that their group affiliation is self-explanatory. It is also okay to leave category(s) blank if you are not sure.

| Person | Group affiliation | Organization/department | Title |
| --- | --- | --- | --- |
| [NAME 1] | [DROP DOWN LIST] |  |  |
| [NAME 2] | [DROP DOWN LIST] |  |  |
| [NAME 3] | [DROP DOWN LIST] |  |  |
| … | … |  |  |

[PAGE BREAK]

The following questions ask about your relationships with the people you listed in the previous section.

1. How **frequently do you typically interact** with each person (in person, by mail, telephone, or Internet) about early childhood obesity in Somerville?

| Person | Daily | Weekly | Monthly | Quarterly | Yearly |
| --- | --- | --- | --- | --- | --- |
| [NAME 1] |  |  |  |  |  |
| [NAME 2] |  |  |  |  |  |
| [NAME 3] |  |  |  |  |  |
| … |  |  |  |  |  |

[PAGE BREAK]

1. How **close is your relationship** with each person on a scale of 1 to 5, where 1 means not close and 5 means very close?

| Person | 1  (not close) | 2  (a little close) | 3  (neutral) | 4  (close) | 5  (very close) |
| --- | --- | --- | --- | --- | --- |
| [NAME 1] |  |  |  |  |  |
| [NAME 2] |  |  |  |  |  |
| [NAME 3] |  |  |  |  |  |
| … |  |  |  |  |  |

[PAGE BREAK]

1. How **influential** is each person in shaping your understanding of issues related to early childhood obesity in Somerville?

| Person | 1  (not influential) | 2  (a little influential) | 3  (neutral) | 4  (influential) | 5  (very influential) |
| --- | --- | --- | --- | --- | --- |
| [NAME 1] |  |  |  |  |  |
| [NAME 2] |  |  |  |  |  |
| [NAME 3] |  |  |  |  |  |
| … |  |  |  |  |  |

[PAGE BREAK]

**Section 4 [DEMOGRAPHICS]**

In this section, you will be asked questions about yourself, including questions about your education, race/ethnicity, and employment.

1. What is your gender?

□ Male

□ Female

□ Other

[PAGE BREAK]

1. What is your age? [SLIDER ON QUALTRICS]

[PAGE BREAK]

1. Are you Hispanic or Latino?

□ Yes

□ No

1. What is your race? *Select all that apply:*

□ American Indian or Alaska Native

□ Asian

□ Native Hawaiian or Other Pacific Islander

□ Black or African American

□ White

□ Some other race (*please specify*): __________

[PAGE BREAK]

1. What is the highest level of education you have completed?

□ Less than high school

□ High school or equivalent

□ Some college (no degree)

□ Associate’s degree

□ Bachelor’s degree

□ Master’s degree

□ Professional or Doctorate degree

[PAGE BREAK]

1. Please select the group you are *most closely* affiliated with when working with children ages 0-5 years. If applicable, please specify the organization or department in the space provided:

□ University __________

□ WIC __________

□ Early education __________

□ Center-based child care __________

□ Home-based child care __________

□ Healthcare __________

□ Health Department __________

□ Community-based organization __________

□ City of Somerville ____________

□ Parent __________

□ Other __________

1. If applicable, please tell us your title for the group/organization/department chosen above, “[INSERT PIPED TEXT]:” ______________________________
2. How many years of experience do you have in this field? [SLIDER ON QUALTRICS]

[PAGE BREAK]

1. Besides “[INSERT PIPED TEXT],” do you have a *secondary* affiliation with another group that works with children ages 0-5 years?

□ Yes

□ No

[IF YES, DISPLAY BELOW]

1. Please select the *secondary* group you are affiliated with when working with children ages 0-5 years. If applicable, please specify the organization or department in the space provided.

□ University __________

□ WIC __________

□ Early education __________

□ Center-based child care __________

□ Home-based child care __________

□ Healthcare __________

□ Health Department __________

□ Community-based organization __________

□ City of Somerville ____________

□ Parent __________

□ Other __________

1. If applicable, please tell us your title for the group/organization/department chosen above, “[INSERT PIPED TEXT]”: ______________________________

[PAGE BREAK]

**END PAGE**

Thank you for participating in the survey! If you have any questions or concerns about this survey or the study in general, please email the principal investigator, [NAME], at [EMAIL] or by telephone at [PHONE NUMBER].
