# Supplementary material for: Development and testing of a novel survey to assess Stakeholder-driven Community Diffusion of childhood obesity prevention efforts
Source: BMC Public Health. 2018 May 31;18:681. doi: 10.1186/s12889-018-5588-1 (PMC5984309; doi:10.1186/s12889-018-5588-1)
Supplement: Supplementary file 4 — Table S3C1-C2. Phase 3 prospective per-item knowledge and engagement reliability results (n = 13 paired responses). Data from test-retest surveys administered online two weeks apart in May 2016: members of the SEA Change and GenR8 Change coalitions in Victoria, Australia. (DOCX 28 kb) [file 12889_2018_5588_MOESM4_ESM.docx]

**Table C1. Phase 3 prospective per-item knowledge reliability results (n=13 paired responses). Data from test-retest surveys administered online two weeks apart in May 2016: members of the SEA Change and GenR8 Change coalitions in Victoria, Australia**

| **#** | **Item** | **Weighted Kappa (κ_w_)** |  |
| --- | --- | --- | --- |
| **Domain 1: The problem of childhood obesity (Problem; 3 items)** | | | |
| 1 | Early childhood obesity is a problem in [community] | 0.26 |  |
| 2 | There are increased health care costs due to medical complications of obesity in early childhood | 0.41 |  |
| 3 | I am familiar with other illnesses and health concerns associated with obesity in early childhood | 0.13 |  |
| **Domain 2: Modifiable determinants of childhood obesity and level of social ecology to address them (Intervention factors; 6 items)** | | | |
| 4 | Preventing obesity early in life is important | 0.00 |  |
| 5 | Certain populations of children are more vulnerable to obesity | 0.26 |  |
| 6 | I am aware of evidence-based strategies that target risk factors related to early childhood obesity | 0.58 |  |
| 7 | I feel confident in listing risk factors related to early childhood obesity | 0.22 |  |
| 8 | I feel confident in listing possible policy changes to combat early childhood obesity in [community] | 0.57 |  |
| 9 | I feel confident in suggesting changes in early childhood settings to prevent early childhood obesity | 0.30 |  |
| **Domain 3: Stakeholders’ roles in the whole intervention, what others are doing, and knowledge of multi-setting components (Roles; 3 items)** | | | |
| 10 | I can play a role in preventing early childhood obesity in [community] | 0.42 |  |
| 11 | I know what is being done in the community to prevent early childhood obesity | 0.61 |  |
| 12 | Obesity preventions strategies should be prioritized and implemented by multiple early childhood providers | 0.48 |  |
| **Domain 4: How to intervene to achieve sustainability (Sustainability; 3 items)** | | | |
| 13 | I know strategies to prevent obesity in early childhood that will be acceptable and appropriate for the community | 0.21 |  |
| 14 | I know strategies to prevent obesity in early childhood that will have the greatest impact in promoting healthy weight | 0.41 |  |
| 15 | I know strategies to prevent obesity in early childhood that can be sustained over time | 0.53 |  |
| **Domain 5: Available resources (Resources; 3 items)** | | | |
| 16 | I know where to find resources related to early childhood obesity prevention | 0.28 |  |
| 17 | I am aware of staff education or training opportunities that include the subject of early childhood obesity prevention | 0.66 |  |
| 18 | I am aware of evaluation and monitoring efforts in [community] that address childhood obesity in young children | 0.38 |  |

**Table C2. Phase 3 prospective per-item engagement reliability results (n=13 paired responses). Data from test-retest surveys administered online two weeks apart in May 2016: members of the SEA Change and GenR8 Change coalitions in Victoria, Australia**

| **#** | **Item** | **Weighted Kappa (κ_w_)** |  |
| --- | --- | --- | --- |
| **Domain 1: Dialogue & mutual learning (7 items)** | | | |
| 1 | I can talk openly and honestly at work or meetings | 0.64 |  |
| 2 | I make an effort to participate in discussions | 0.42 |  |
| 3 | I listen to colleagues when someone expresses a concern | 0.45 |  |
| 4 | I am attentive to what colleagues say when they speak | 0.58 |  |
| 5 | I share my ideas and suggestions whether or not colleagues agree with my input | 0.51 |  |
| 6 | I can openly discuss problems and issues | 0.25 |  |
| 7 | I work with colleagues to develop the best possible approach to our work | 0.30 |  |
| **Domain 2: Flexibility (3 items)** | | | |
| 8 | I am willing to make compromises related to my work in early childhood obesity prevention | 0.47 |  |
| 9 | I work to come up with solutions that satisfy all colleagues | 0.25 |  |
| 10 | I respect different points of view from colleagues | 0.05 |  |
| **Domain 3: Influence & power (2 items)** | | | |
| 11 | I influence decisions that affect early childhood obesity prevention efforts in the community | 0.16 |  |
| 12 | I influence the policies and actions of community-based early childhood obesity prevention | 0.36 |  |
| **Domain 4: Leadership (10 items)** | | | |
| 13 | I am motivated to prevent early childhood obesity | 1.00 |  |
| 14 | My passion and enthusiasm for early childhood obesity prevention motivates others | 0.43 |  |
| 15 | I establish positive relationships with community members with whom my colleagues want to engage and mobilize | 0.39 |  |
| 16 | I have good skills for working with other people and organizations | -0.18 |  |
| 17 | I lead by example | 0.54 |  |
| 18 | I encourage colleagues to express their opinions and thoughts | -0.16 |  |
| 19 | I emphasize the importance of having a collective sense of mission | 0.29 |  |
| 20 | I provide leadership and guidance in maintaining relationships among colleagues | -0.12 |  |
| 21 | I advocate strongly for my own opinions and agendas | 0.13 |  |
| 22 | I do not give up when faced with challenges | 0.15 |  |
| **Domain 5: Trust (3 items)** | | | |
| 23 | I trust others involved in early childhood obesity prevention efforts | 0.06 |  |
| 24 | People involved in early childhood obesity prevention efforts trust me | 0.09 |  |
| 25 | I try to promote a climate of collaboration and trust | 0.09 |  |
